# Modulation of inflammation in transgenic models of Alzheimer’s disease

**DOI:** 10.1186/1742-2094-11-25

**Published:** 2014-02-03

**Authors:** Amy M Birch, Loukia Katsouri, Magdalena Sastre

**Affiliations:** 1 Division of Brain Sciences, Imperial College London, London W12 0NN, UK

**Keywords:** Inflammation, Microglia, Astrocytes, Amyloid, Tau, Transgenics, Anti-inflammatory

## Abstract

Over the past decade the process of inflammation has been a focus of increasing interest in the Alzheimer’s disease (AD) field, not only for its potential role in neuronal degeneration but also as a promising therapeutic target. However, recent research in this field has provided divergent outcomes, largely due to the use of different models and different stages of the disease when the investigations have been carried out. It is now accepted that microglia, and possibly astrocytes, change their activation phenotype during ageing and the stage of the disease, and therefore these are important factors to have in mind to define the function of different inflammatory components as well as potential therapies. Modulating inflammation using animal models of AD has offered the possibility to investigate inflammatory components individually and manipulate inflammatory genes in amyloid precursor protein and tau transgenics independently. This has also offered some hints on the mechanisms by which these factors may affect AD pathology. In this review we examine the different transgenic approaches and treatments that have been reported to modulate inflammation using animal models of AD. These studies have provided evidence that enhancing inflammation is linked with increases in amyloid-beta (Aβ) generation, Aβ aggregation and tau phosphorylation. However, the alterations on tau phosphorylation can be independent of changes in Aβ levels by these inflammatory mediators.

## Background

During the last 10 years, interest in research related to Alzheimer’s disease (AD) and inflammation has grown significantly. Ageing is the greatest risk factor for development of AD and this is thought, in part, to be due to enhanced chronic inflammation associated with increasing age [1]. In addition, it has been recognised that amyloid-beta (Aβ) is able to initiate an inflammatory response, which implicates the activation of microglia and the recruitment of astrocytes, and therefore the release of cytokines, chemokines, reactive oxygen species and neurotoxic products that have been involved in neuronal and synaptic damage [2]. Mice expressing mutant amyloid precursor protein (APP) or tau do not present significant neuronal loss; therefore, it has been theorised that the addition of the inflammatory component of AD would result in a more appropriate model to investigate the disease. Due to the well-documented changes in inflammatory markers detected in the AD brain and the inflammatory risk factors associated with the disease, targeting these processes has become increasingly attractive and the use of anti-inflammatory drugs has shown potential as a preventive treatment. In this review we aim to describe different genetic and drug manipulations that have been carried out in AD animal models and that have allowed the identification of mechanisms by which inflammation is a relevant factor to incorporate as a hallmark for AD pathology.

## Modelling Alzheimer’s disease

It is now widely accepted that Aβ induces glial activation and therefore mouse models of Alzheimer’s disease overexpressing the human APP with familial AD mutations, such as the Tg2567 and the APP23 (both carrying the Swedish mutation, APP_SWE_), have been shown to present microglial and astrocytic activation [3,4]. In addition, their brains display enhanced levels of cytokines such as TNFα, IFNγ, IL-1β, IL-1α, chemoattractant protein-1, cyclooxygenase (COX)-2 and complement component 1q [2,3,5]. The characterisation of inflammatory processes in the APPV717I mouse model has demonstrated that focal glial activation occurs before amyloid plaque formation, already at 3 months of age [6], in parallel with decreased LTP (long-term potentiation) [7]. However, there have been some issues on how well these mouse models mimic human pathology because they do not show the robust tauopathy and neuronal death that is evident in the human disease unless additional human transgenes such as tau are added [8]. The triple-transgenic model (3×Tg-AD), which harbours APP_SWE_, presenilin-1 (PS1_M146V_) mutation, and tau mutation (tau_P301L_), offers the advantage of developing progressive plaque deposition and tangle formation together with microglial activation and an upregulation of the pro-inflammatory cytokine TNFα and chemokine MCP-1 (CCL2), although this is limited to the entorhinal cortex [9].

Mouse models of tauopathy such as the P301S tau transgenic (Tg) mouse also exhibit neuroinflammatory changes, exemplified by the co-localisation of aggregated tau, IL-1β and COX-2, reactive astrocytosis and the accumulation of activated microglial cells around tau-positive neuronal cells [10]. Interestingly, microglial activation precedes tangle formation in 3-month-old P301S Tg mice [11], and therefore neuroinflammation has been proposed to be the link between Aβ deposition and the formation of neurofibrillary tangles.

Perhaps one of the more promising advances in modelling AD comes from the development of a rat model that coexpresses the human APP_SWE_ and PS1ΔE9 [12]. These TgF344-AD rats develop all the hallmarks typically seen in Tg-AD mice (age-dependent cerebral amyloidosis, glial activation, and memory impairments) but additionally exhibit tauopathy and neuronal death and therefore more closely mimic human AD pathology. This model provides further support for the amyloid cascade hypothesis and it is hoped that it will provide a next step in translational therapeutic studies for AD and further enhance understanding of the basic neuropathology and the underlying causes of this disease.

Activated glial cells can be imaged *in vivo* in animal models of AD using positron emission tomography. The development of tracers for activated microglia is based on the observation that the peripheral benzodiazepine receptor is upregulated in activated microglia. Ligands such as [^11^C](R)-PK11195 bind to this receptor, also known as the translocator protein (TSPO). A significant age-dependent increase in specific [^3^H](R)-PK11195 binding was demonstrated in a transgenic mouse model of AD by autoradiography (TASTPM: APP_sw_xPS1_M146V_; [13]). However, [^11^C]-(R)-PK11195 positron emission tomography could not demonstrate differences between wild-types and transgenic APP/PS1 mice [14]. This tracer has some limitations, such as high non-specific binding and high binding to plasma proteins. These issues have consequently led to the development of new radiotracers targeting TSPO including [^18^F]-PBR111, ^11^C-radiolabelled and ^18^F-radiollabeled versions of PBR06 and PBR28 as well as [^18^F]-FEPPA [15]. In fact, radiolabelling of TSPO with [^11^C]AC-5216 was linearly proportional to the amount of phospho-tau immunolabelling in transgenic PS19 mice carrying the P301S tau mutation [16]. The results of that study indicated that TSPO immunoreactivities are more likely to be associated with neurofibrillary tangles rather than Aβ deposits.

## Modulation of inflammatory processes in models of Alzheimer’s disease

### Modulation in amyloid precursor protein transgenic models

Genetic manipulation of several immune and inflammatory pathways in mouse models of AD has been carried out during the past decade to explore how increasing or decreasing neuroinflammation may affect AD progression (see Table 1). Unfortunately, most of these reports have focused only on the effect on amyloid deposition and there is a general lack of cognitive and longitudinal live imaging studies. These investigations have provided some indications to potential mechanisms by which inflammation may trigger changes in AD pathology. However, there has been some variability in the results obtained from these studies, which are largely dependent upon in which transgenic mouse model the studies have been carried out. For example, deletion of inducible nitric oxide synthase (iNOS) in an APP/PS1 background resulted in different outcomes on Aβ load compared to iNOS knockout in the Tg2576 mouse model [17,18]. In general it is expected that overexpression of pro-inflammatory mediators will enhance progression of the disease and therefore treatments should follow an anti-inflammatory approach. For example, blocking signaling of the pro-inflammatory cytokines IL-12 and IL-23 via ablation of the common subunit p40 in APP/PS1 mice has been shown to reduce glial activation and amyloid burden [19]. Furthermore, IFNγ signaling loss in APP mice knockout for IFNγ receptor type I (GRKO mice) reduced gliosis and amyloid plaques in Tg2576 mice [20]. Interestingly, a significant reduction in the number of BACE1-positive astrocytes was seen in APP/GRKO mice as compared with APP littermates. In line with this, deletion of TNFRI in APP23 mice has been reported to reduce BACE1 protein levels and activity as well [21]. These studies in animal models support our *in vitro* observations, which showed that inflammation enhances BACE1 expression [22,23].

**Table 1:** Modulation of inflammatory mediators in Alzheimer’s disease mouse models

**AD mouse model**	**Genetic manipulation**	**Effect on Alzheimer-like pathology**	**Reference**
APP23^1^	TNF-RI−/−	↓Aβ, ↓amyloid plaques, ↓microglial activation, ↓BACE1, ↓neuronal loss, ↑memory	[21]
3xTg-AD^2^	TNF-RI/RII−/−	↑Aβ, ↑amyloid plaques, ↑PHF, ↓IBA1, ↓microglial phagocytosis, ↓LTP	[24]
3xTg-AD^2^	TNFα−/−	↑Aβ, ↔memory improvement	[25]
3xTg-AD^2^	TgIL-1β^XAT^	↓Aβ, ↑p-tau, ↑glial activation	[26]
APP/PS1^3^	TgIL-1β^XAT^	↓Aβ, ↑glial activation, ↑cytokines	[27]
APP/PS1^3^	TgIL-1β^XAT^	↓Aβ, ↓amyloid plaques	[28]
APP/PS1^4^	IL-12α−/−	↓Aβ	[19]
APP/PS1^4^	IL-12β−/−	↓Aβ, ↓glial activation	[19]
APP/PS1^4^	IL-23−/−	↓Aβ	[19]
PDGF-APP_SweInd_ line J9^5^	GFAP-TGFβ1	↓Aβ, ↑cerebrovascular Aβ, ↑glial activation	[29]
PDAPP^6^	GFAP-TGFβ1	↑cerebrovascular Aβ, ↑CAA, ↑perivascular astrocytes	[30]
Tg2576^7^	CD11c-DNR(TGF-β)	↓Aβ, ↓memory impairment, ↓CAA	[31]
Tg2576^7^	IFNγRI−/−	↓Aβ, ↓glial activation	[20]
APP/PS1^3^	Mrp14−/−	↓Aβ, ↓BACE1, ↓cytokines, ↑microglial activation, ↑Aβ phagocytosis	[32]
Tg2576^7^	NOS2−/−	↑Aβ,↑p-tau, ↑neuronal death	[17]
APP/PS1^3^	NOS2−/−	↓Aβ, ↓plaques, ↑LTP, ↑memory	[18]
APP/PS1^3^	NOS2−/−	↑IDE	[33]
Tg-SwDI/B^8^	NOS2−/−	↔Aβ, ↑p-tau, ↑CAA, ↑neuronal loss, ↑memory impairment	[34]
PDGF-APP_SweInd_ line J9^5^	PDGF-RAGE	↑Aβ, ↑glial activation, ↓LTP	[35]
PDGF-APP_SweInd_ line J9^5^	GFAP-α1-ACT	↑Aβ	[36]
PDAPP^6^	GFAP-α1-ACT	↑Aβ, ↑plaques	[37]
PDAPP^6^	GFAP-α1-ACT	↑p-tau	[38]

^1^hAPP Swedish mutation under the murine Thy1.2 promoter. ^2^hAPP Swedish, hPS1 knock-in with M146V mutation, htau P301L mutation. APP and Tau are under the Thy1 promoter. ^3^hAPP Swedish and hPS1dE9 mutations under the murine Thy1.2 promoter. ^4^hAPP Swedish and hPS1 L166P mutations under the murine Thy1 promoter. ^5^hAPP Swedish and Indiana mutations under the PDGF promoter. ^6^hAPP Indiana mutation under the PDGF promoter. ^7^hAPP Swedish mutation under the hamster prion promoter. ^8^hAPP Swedish, Dutch and Iowa mutations under the murine Thy1.2 promoter. Aβ, amyloid-beta; ACT, antichymotrypsin; AD, Alzheimer’s disease; APP, amyloid precursor protein; CAA, cerebral amyloid angiopathy; GFAP, glial fibrillary acidic protein; IBA, ionized calcium binding adaptor molecule-1; IDE, insulin degrading enzyme; IFN, interferon; IL, interleukin; LTP, long-term potentiation; NOS, nitric oxide synthase; PDAPP, amyloid precursor protein under control of platelet-derived growth factor promoter; PDGF, platelet-derived growth factor; PHF, Paired helical filament; RAGE, Receptor for Advanced Glycation End; Tg, transgenic; TGF, transforming growth factor; TNF, tumor necrosis factor.

Another potential way by which inflammation may contribute to AD pathology is by increasing Aβ aggregation. Nitration of Aβ has been shown to accelerate its aggregation and was detected in the core of Aβ plaques of APP/PS1 mice and AD brains. Studies carried out in nitric oxide synthase (NOS)2 knockout mice have shown strongly decreased 3NTyr(10)-Aβ, overall Aβ deposition and cognitive dysfunction in APP/PS1 mice [18].

A recently studied and significant factor in immune response is the NLRP3 inflammasome, which is a multiprotein oligomer consisting of caspase 1, PYCARD, NALP and sometimes caspase 5. It is upregulated in response to the stimulation of macrophages with pathogen-associated molecule patterns. APP/PS1 mice crossed with NLRP3−/− mice have rescued spatial memory, synaptic plasticity and a reduction in Aβ load when compared with age-matched APP/PS1 mice. These changes were associated with an increase in microglial phagocytic activity and increased insulin degrading enzyme [39]. APP/PS1 mice deficient in NLRP3 show increased M2 phenotype markers: FIZZ1, arginase-1, and IL-4, with reduced NOS2 expression. Complementary results were also shown with deletion of caspase 1, an important effector enzyme, in APP/PS1 mice [39].

Yet, intriguingly, many studies that induce an inflammatory state by administration of lipopolysaccharide (LPS) or IL-1β lead to a decrease in Aβ burden. This effect has been associated with enhanced microglial activation and subsequent Aβ clearance [27,28,40-42]. This is a seemingly artificial method of activation of microglia, however, as acute administration of these strongly activating factors does not mimic the chronic inflammation shown in AD and, as IL-1β and LPS induce memory impairments in rodents [43,44], they could never be thought of as a viable therapy.

In summary, studies in APP models have shown that inflammation may potentiate AD pathology in APP transgenic mice by increasing Aβ generation, aggregation and by affecting its clearance.

### Modulation in tau models

There is a scarcity of studies directly assessing the effect of inflammation in tau models of AD. The few that have been conducted have led to some intriguing results that suggest there may be immune responses to APP processing and tau hyperphosphorylation. While APP or APP/PS1 models do not develop neurofibrillary tangles, they do show increased tau phosphorylation [45,46]. Products of inflammation, such as pro-inflammatory cytokines, can change the substrate specificity of kinases/phosphatases leading to tau phosphorylation at pathological sites [47].

Unlike the increases in Aβ pathology shown in PDAPP J20 [48], suppression of inflammation by overexpressing the complement inhibitory factor sCrry in P301L tau Tg mice resulted in reduced tau pathology [49]. This suggests that acute activation of the complement activation pathway is detrimental in tau mice. The 3×Tg-AD mouse model has also been used to test the effect of manipulating inflammation on tau pathology. Acute activation of the immune response in 3×Tg-AD mice by LPS treatment induced tau hyperphosphorylation via a Cdk5-dependent mechanism [50]; however, no changes were detected in amyloid pathology. In line with this, viral infection-induced acute or chronic inflammation in 3×Tg-AD mice significantly exacerbated tau pathology and led to impairments in spatial memory. In this case, tau phosphorylation was increased via a glycogen synthase kinase-3β-dependent mechanism [51]. Other approaches to induce inflammation in the brain of the 3×Tg-AD mice such as by controlled cortical impact traumatic brain injury caused acute intra-axonal Aβ accumulation and increased phospho-tau [52]. Interestingly, and in contrast to that which is observed for transgenic mice overexpressing only APP, IL-1β overexpression in 3×Tg-AD mice resulted in increased tau phosphorylation, associated with higher p38 MAPK and GSK3β activity with reductions in Aβ load [26].

However, other studies have shown controversial results in this 3×Tg-AD model, reporting reduced tau phosphorylation after delivery of IFNγ (rAAV1-IFNγ) via recombinant adeno-associated virus vector [53]. In addition, disruption of TNFα signaling in 3×Tg-AD exacerbated amyloid and tau pathology [24,54].

With these last few exceptions, the results of modulation of inflammation in tau transgenics suggest that increased neuroinflammation leads to enhanced phosphorylation of tau, and this effect is not necessarily dependent on increased Aβ generation.

## Glial modulation in Alzheimer’s disease models

### Microglia manipulation in Alzheimer’s disease models

The microglial/macrophage response is a key mediator of the immune response in the brain. Microglia can be activated following exogenous or endogenous stimulation by a variety of receptors. Stimulation of these receptors can induce activation of microglia into a ‘classical (M1)’ or ‘alternative (M2)’ phenotype. That microglia play a significant role in eliciting inflammation and clearing toxic products and damaged tissue cannot be disputed, but their direct role in disease progression is unclear. Near complete ablation of microglia, by crossing either APP23 or APP/PS1 mice with CD11b-TK mice, did not show differences in plaque formation and only a very small reduction in diffuse Aβ in the APP23/CD11b-TK model [55], suggesting more subtle approaches to study their role are necessary.

A number of recent reviews have highlighted the current literature trends and debated the seemingly contradictory results relating to microglial involvement in AD [56-58]. The activation state of microglia and their ability to phagocytose and clear amyloid in the brain seems to be a significant, but contentious, factor. Microglia and macrophages express a number of different receptors that can promote phagocytosis and clearance of Aβ that have been targeted. These include complement receptors, scavenger receptors, and cytokine/chemokine receptors that are associated with pathogen recognition (Table 2). These data can often seem incompatible and contradictory in many cases and yet yield some significant therapeutic targets and emphasize the multi-faceted and heterozygous nature of microglial response in AD from the beginning of the disease throughout its progression. Specific manipulation of signaling factors associated with a shift to the M2 phenotype is reported to promote clearance of Aβ and ameliorate other symptoms, as microglia exhibit a more anti-inflammatory, phagocytic phenotype. For example, suppression of fractalkine signaling, a negative regulator of microglial activation, is successful in reducing amyloid plaque burden and neuronal loss [59-62]. In mouse models of other neurodegenerative disease such as Parkinson’s disease or ALS (amyotrophic lateral sclerosis), lack of CX3CR1 causes widespread neuron loss [63], suggesting that the microglial activation profile seen here is an AD-specific effect. However, as APP mouse models do not exhibit significant neuron loss it is difficult to conclude if this is a tau-specific effect or relevant to human AD.

**Table 2:** Modulation of glia in Alzheimer’s disease mouse models

**AD mouse model**	**Genetic manipulation**	**Effect on Alzheimer-like pathology**	**Reference**
APP/PS1^1^	Scara1−/−	↑Aβ, ↑mortality, ↓IDE, ↓Neprilysin	[64]
PDAPP_SweInd_ line J20^2^	Scarb1−/−	↑amyloid plaques, ↑CAA, ↔glial activation, ↑memory impairment	[65]
APP/PS1^3^	CD11b-TK	↔Aβ, ↔amyloid plaques, ↑GFAP, ↓Iba1	[55]
APP23^4^	CD11b-TK	↓Aβ, ↓Iba1, ↔amyloid plaques	[55]
PDAPP_SweInd_ line J20^2^	CxCR3-GFP ki	↔Aβ, ↑microglial activation, ↑IL-6, ↑TNF-α, ↑p-tau, ↑memory impairment	[62]
TgCRND8^5^	CxCR3-GFP ki	↓Aβ, ↓amyloid plaques, ↑ microglial phagocytosis, ↑microglial proliferation	[59]
APP/PS1^3^	CxCR3-GFP ki	↓Aβ, ↓amyloid plaques, ↓microglia, ↑ microglial phagocytosis	[60]
R1.40^6^	CxCR3-GFP ki	↓Aβ, ↓amyloid plaques	[60]
htau^7^	CxCR3-GFP ki	↑p-tau, ↑Gallyas-positive dystrophic neurites, ↓Iba1, ↑microglial activation (CD68^+^ and CD45^+^)	[66]
3xTg-AD^8^	CxCR3-GFP ki	↓neuronal loss	[61]
Tg2576^9^	Ccr2−/−	↑Aβ, ↓NEP	[67]
APP/PS1^10^	Ccr2−/−	↑soluble Aβ, ↑microglial activation, ↑memory impairment	[68]
APP/PS1^10^	NSE-COX2	↑Aβ, ↑PGE2	[69]
Tg2576^9^	C1q−/−	↔Aβ, ↓glial activation, ↑neuronal degeneration	[70]
Tg2576^9^	C1q−/−	↔Aβ, ↓glial activation, ↓loss of synaptic markers	[71]
APP/PS1^11^	C1q−/−	↔Aβ, ↓glial activation	[71]
Tau_P301L_ line JNLP3^12^	sCrry	↑p-tau	[49]
Tg2576^9^	CD40L−/−	↓p-tau	[72]
Tg2576^9^	CD40L−/−	↓Aβ, ↓glial activation	[73,74]
APP/PS1^11^	CD40L−/−	↓Aβ, ↓glial activation	[73]
APP/PS1^1^	Nlrp3−/−	↓Aβ, ↓plaques, ↓IL-1β, ↓iNOS, ↑LTP, ↑spatial memory, ↑IDE	[39]
PDAPP_SweInd_ line J20^2^	C3−/−	↑Aβ, ↑amyloid plaques, ↑glial activation, ↑neuronal loss	[75]
APP/PS1^1^	CD14−/−	↓Aβ, ↓amyloid plaques, ↓CD45^+^ activated microglia	[76]
APP/PS1^1^	CD33−/−	↓Aβ, ↓plaques	[77]
Tg2576^9^ (before plaque onset)	CD36−/−	↔Aβ, ↔ROS	[78]
Tg2576^9^ (old mice)	CD36−/−	↓Aβ_40_, ↓CAA, ↑cognitive performance	[79]
APP/PS1^1^	CD45−/−	↑Aβ, ↑amyloid plaques, ↑inflammatory microglia, ↑TNF-α, ↑IL-1β, ↑neuronal death	[80]
APP/PS1^3^	IRAK4^KI/KI^	↓Aβ, ↓amyloid plaques, ↓glial activation, ↑PPARγ, ↑IDE, ↑IFNγ, ↓iNOS	[81]
APP/PS1^1^	TLR4^Lps-d^	↑Aβ, ↑amyloid plaques	[82]
APP/PS1^1^	TLR4^Lps-d^	↑CD11b^+^ microglia, ↑GFAP	[83]
APP/PS1^1^	TLR4^Lps-d^	↑Aβ, ↑ amyloid plaques, ↓microglial activation, ↑cognitive impairment	[84]
APP/PS1^1^	MyD88−/−	↓Aβ, ↓amyloid plaques, ↓CD11b^+^, CD45^+^ microglia	[85]
APP/PS1^10^	MyD88+/−	↓amyloid plaques, ↑soluble Aβ, ↓IL-1β	[86]
APP/PS1^10^	TLR2−/−	Delayed plaque formation, ↑Aβ, ↑TGF-β, ↑memory impairment	[87]
Tg2576^9^	GFAP-MCP1	↑Aβ, ↑microglial activation	[88]
APP/PS1^1^	GFAP−/−Vim−/−	↑Aβ, ↑amyloid plaques, ↑neurotic dystrophy, ↓activated astrocytes, ↑microglia,	[89]

^1^hAPP Swedish and hPS1dE9 mutations under the murine Thy1.2 promoter. ^2^hAPP Swedish and Indiana mutations under the PDGF promoter. ^3^hAPP Swedish and hPS1 L166P mutation under the Thy1 promoter. ^4^hAPP Swedish mutation under the murine Thy1.2 promoter. ^5^hAPP Swedish and Indiana mutations under the hamster prion promoter. ^6^YAC with 300Kb hAPP gene with the Swedish mutation. ^7^*Mapt−/−* mice crossed with Tg(MAPT)8cPdav that contains the whole 5′-flanking and exons 1–14 of the h*MAPT* gene. ^8^hAPP Swedish mutation, hPS1 knock-in with M146V mutation, htau P301L mutation. hAPP and hTau are under the Thy1 promoter. ^9^hAPP Swedish mutation under the hamster prion promoter. ^10^hAPP Swedish mutation and hPS1 with the A246E mutation both under the mouse prion promoter. ^11^Tg2576 (hAPP Swedish mutation) crossed with hPS1 with the M146L mutation. ^12^hTau with the P301L mutation under the mouse prion promoter. Aβ, amyloid-beta; AD, Alzheimer’s disease; APP, amyloid precursor protein; CAA, cerebral amyloid angiopathy; GFAP, glial fibrillary acidic protein; GFP, green Fluorescent Protein; IBA, ionized calcium binding adaptor molecule-1; IDE, insulin degrading enzyme; IFN, interferon; IL, interleukin; iNOS, inducible nitric oxide synthase; LTP, long-term potentiation; MyD88, myeloid differentiation primary response protein 88; NSE-COX2, neuron-specific enolase-cyclooxigenase-2; PDAPP, amyloid precursor protein under control of platelet-derived growth factor promoter; PDGF, platelet-derived growth factor; PGE2, prostaglandin E2; PHF, Paired helical filament; PPAR, peroxisome proliferator-activated receptor; RAGE, Receptor for Advanced Glycation End; ROS, reactive oxygen species; Scar, scavenger receptor; Tg, transgenic; TGF, transforming growth factor; TLR, Toll-like receptor; TNF, tumor necrosis factor.

In addition, targeting of the phagocytic phenotype of microglia has shown some promising results in AD mouse models. The complement pathway has been extensively studied in relation to AD and reports suggest that upregulating complement factors may target inflammatory processes by promoting migration and phagocytosis of inflammatory cells [48,71,75].

Microglia and macrophages express a number of receptors that can promote clearance of Aβ, such as scavenger receptor class A1 (*Scara1*) and class BI (*Scarb1*). Knockout models for *Scarb1*[65] and *Scara1*[64] have shown alterations in Aβ load.

Additionally, Toll-like receptors (TLRs) and their co-receptors including MD-2, CD14, and CD36 [90] are of great importance for the recognition of pathogens in the body and participate in the response of microglial cells to fibrillar forms of Aβ [91]. Deletion of CD14, which acts as a co-receptor for LPS along with TLR2 and TLR4, in APP/PS1 mice reduced total microglial numbers, in particular CD45-positive microglia, attenuated AD pathology whilst also increasing the expression of TNF-α and IL-10, suggesting an induction of a shift of activation of microglia towards the M2b state [76]. On the other hand, TLR2 deficiency accelerated spatial and contextual memory impairments, which correlated with increased levels of Aβ(1–42) and transforming growth factor-β in the brain of APP/PS1 mice [87]. An essential adaptor protein for all TLR signaling, with the exception of TLR3, is the myeloid differentiation primary response protein 88 (MyD88). Decreasing the expression of MyD88 in APP/PS1 mice led to exacerbation of spatial memory deficits, increases in Aβ, reduced expression of the fractalkine receptor CX3CR1 and increased levels of APOE (Apolipoprotein E) together with reduced astrocyte and microglial activation [85,86]. These data indicate that TLR2 and TLR4 may be involved in Aβ clearance *in vivo* and hence provide neuroprotection in AD [92]. They also suggest that targeting specific glial activation states may prove fruitful in future clinical studies.

CD33 gene and TREM2, which are expressed in microglia, have been recently identified as genetic risks factors for AD [93-96]. It was reported that CD33 is able to inhibit the uptake and clearance of Aβ42 in microglial cell cultures. This was confirmed by *in vivo* results showing that brain levels of insoluble Aβ42 as well as amyloid plaque burden were markedly reduced in APP(Swe)/PS1(ΔE9)/CD33(−/−) mice. Therefore, CD33 inactivation appears to mitigate Aβ pathology [77]. On the other hand, hypothesizing that the TREM2 risk variants impair TREM2 function, these new genetic studies suggest that reduced function of TREM2 causes reduced phagocytic clearance of amyloid proteins or cellular debris and thus impairs a protective mechanism in the brain [94,96].

There are a number of studies that attribute the clearance of amyloid in mouse models to infiltrating monocytes or perivascular macrophages [97-100]. This is due to the evidence showing a reduced efficiency of microglia with age [101] and bacterial and viral infections [102]. However, the role of these peripheral monocytes in neurodegeneration remains unclear. One important aspect is the contribution of monocytes to resident macrophages, which is highly tissue-dependent and has been shown not to be relevant for brain microglia. However, recently it was suggested that, irrespective of their origin, macrophages/microglia can self-renew by local proliferation similar to that of stem cells [103]. In fact, in animal models of prion disease it has been demonstrated that microglial proliferation is a major component in the evolution of chronic neurodegeneration [104].

Many models that show peripheral monocytic infiltration use whole body irradiation which damages the blood–brain barrier itself, induces peripheral immune activation and can facilitate infiltration. Using this approach, it was recently published that microglia-depleted brain regions of CD11b-TK transgenic mice are repopulated with new Iba1-positive cells within 2 weeks, creating a niche for myeloid cells [105]. However, using the technique of parabiosis (in which two mice share vasculature), GFP (Green Fluorescent Protein) -labelled monocytes from one mouse are not seen to infiltrate the brain of the other mouse, except following irradiation and bone marrow transplantation, which would suggest a pre-existing disease state is necessary in the brain for significant infiltration to occur [102,106]. In line with this, recent data provide strong evidence that the engraftment of myeloid cells in the brain parenchyma of AD transgenic mice does not occur normally during disease progression, but requires prior central nervous system conditioning to sufficiently attract bone marrow cells [102]. These studies also highlight the importance of the chemokine receptor CCR2 in monocyte migration as the infiltrating cells following irradiation are characterized as CCR2^+^. Interestingly, deletion of CCR2 in Tg2576 mice increased Aβ accumulation and reduced microglial recruitment into the brain, in particular phagocytic macrophages [67]. In agreement with this, another study showed that restriction of CCR2 deficiency to perivascular myeloid cells drastically impaired Aβ clearance and amplified vascular Aβ deposition, while parenchymal plaque deposition remained unaffected [102].

Furthermore, inflammatory IFNγ-secreting Th1 cells and IL-17-secreting Th17 cells have been shown to infiltrate the brain of older APP/PS1 mice [107], supporting the observation of infiltrating T cells in the brain of AD patients [108]. However, the role of these cells in the AD brain is still unknown.

### Manipulation of astrocytes in animal models of Alzheimer’s disease

Astrocytes are becoming increasingly recognized as having key immune functions in the brain, and their role in Alzheimer’s disease progression has recently been investigated. Whilst currently falling behind the number of studies that are published assessing microglial function in AD, it is clear that astrocytes have a significant role to play in AD and therefore warrant significant future research.

Attenuation of astrocytic activation via deletion of *GFAP* and *vimentin* in APP/PS1 mice exacerbated amyloid plaque load independent of APP processing and Aβ production [89], suggesting that astrocytes are important in amyloid clearance. Yet a previous study has shown that blocking astrocyte activation via AAV-*Gfa2* vectors in APP/PS1 mice also attenuates microglia activation, improves cognitive and synaptic function and reduces amyloid load [109]. However these mice were analyzed at a considerably older age (16 to 18 months) when compared with the more recent study (8 to 12 months) which suggests that there may be a significant timing factor involved in targeting the immune response in AD.

Whether astrocytes are promoting amyloid clearance or exacerbating deposition is in debate; α_1_-antichymotrypsin (α_1_-ACT), an acute-phase protein that is overexpressed by activated astrocytes surrounding the amyloid plaques in human AD brains, has been proven to promote Aβ fibrillization. Confirming this, overexpression of a human transgene by astrocytes in the PDGF-APP_SweInd_ J9 or PDAPP mouse model promoted Aβ deposition and plaque formation [36,37]. It also affected tau phosphorylation and p-tau was increased both in single transgenic GFAP-α_1_-ACT and in APP-GFAP-α_1_-ACT mice [38].

## Anti-inflammatory therapy

### Non-steroidal anti-inflammatory drugs

Many inflammatory pathways have been implicated in AD, yet these pathways are not sufficiently well delineated to define those processes and targets that may be pathogenic as opposed to those that may be protective. The finding that treatment with non-steroidal anti-inflammatory drugs (NSAIDs) is associated with a reduced risk and age of onset of AD reinforces the hypothesis that modulating inflammation could have therapeutic efficacy. The beneficial effects of NSAIDs have also been associated with reductions in Aβ generation, since experiments *in vitro* and in AD models indicate that certain NSAIDs are able to decrease Aβ levels, plaque size and tau phosphorylation [110,111].

The mechanism by which NSAIDs are protective has yielded controversial results. The initial hypothesis was that NSAIDs may affect Aβ aggregation [112,113]. Following this, it was suggested that a subset of NSAIDs was affecting the γ-secretase cleavage site and the ratio Aβ40/42 [114,115]. Some recent studies have shown that treatment of AD mice with a novel NSAID derivative, CHF5074, which has a more selective action on γ-secretase, resulted in modulation of Aβ42 production without affecting C-terminal APP or Notch processing [116-118]. Chronic treatment in Tg2576 mice ameliorated memory deficits and loss of dendritic spine density together with a reduction in Aβ load, activated microglia and neuronal cell death [119]. Another potential target of NSAIDs is COX-1 [120]. It was recently reported that treatment of 3×Tg-AD mice with the COX-1 selective inhibitor SC-560 improved spatial learning and memory, and reduced amyloid deposits and tau hyperphosphorylation. SC-560 also reduced glial activation and brain expression of inflammatory markers [121]. Certain NSAIDs are also agonists for peroxisome proliferator-activated receptor (PPAR)γ and have been shown to reduce BACE1 [22,122]. However, PPARγ activation can affect the transcription of other proteins involved in AD as well (see section below).

However, clinical trials have failed to reproduce the beneficial effects of NSAIDs in AD patients. The success of NSAIDs clinically is likely to be dependent on the stage of the disease at which the medication is started as well as the duration of the treatment [111], since their benefit seems to be towards a preventive effect rather than a therapeutic option. Interestingly, clinical trials with anti-inflammatory drugs such as trifusal in MCI (mild cognitive impairment) patients have shown a significant lower rate of conversion to dementia that is likely to be clinically relevant [123].

### Peroxisome proliferator-activated receptor-γ agonists

PPARγ is a nuclear receptor that regulates the transcription of pro-inflammatory genes, such as IL1β and iNOS. Activation of PPARγ is therefore able to inhibit the inflammatory response, and acute and chronic treatment with the PPARγ agonist pioglitazone in APPV717I and Tg2576 mice resulted in a reduction in the number of activated microglia [122,124]. In addition, our group found that PPARγ activators decrease total Aβ levels under inflammatory conditions by affecting BACE1 transcription [6,22,23]. On the other hand, it was shown in neuronal cells that ibuprofen is able to suppress RhoA activity through PPARγ activation, promoting neurite elongation [125]. Therefore PPARγ activation could be beneficial in AD at several levels.

Other groups have suggested that PPARγ may affect Aβ clearance and degradation. It was recently demonstrated that PPARγ activation induces *lxrα, apoe*, and *abca1* expression, promoting Aβ clearance by both microglia and astrocytes [126]. Furthermore, PPARγ can stimulate Aβ phagocytosis by the upregulation of scavenger receptor CD36 expression. It has also been shown that combined treatment with agonists for the heterodimeric binding partners of PPARγ, the retinoid X receptors (RXRs), showed additive enhancement of the Aβ uptake that was mediated by RXRα activation [127].

Treatment with PPAR agonists has also shown benefits in tau models. Treatment with the pan-PPAR agonist bezafibrate significantly decreased tau hyperphosphorylation and caused behavioural improvement, as evidenced by reduced hyperactivity and disinhibition in P301S mice [128]. In addition, 3×Tg-AD mice treated with pioglitazone for 4 months showed improved learning, decreased hippocampal Aβ and tau deposits, and enhanced short- and long-term plasticity [129].

Clinical trials with PPARγ activators have been more successful than those with NSAIDs. A randomised study with pioglitazone (a typical PPARγ agonist) showed significantly increased memory scores in treated patients [130]. Another PPARγ agonist, rosiglitazone, has been trialled with inconsistent results, due to its lack of permeability in the brain and its differential effects depending on the APOE (Apolipoprotein E) ϵ4 genotype [131].

### Minocycline

Minocycline, a tetracycline derivative, has potent anti-inflammatory, anti-apoptotic, and neuroprotective properties. In many cases, the neuroprotective properties of minocycline have been attributed to inhibition of caspases. In primary cortical neurons, minocycline was shown to reduce caspase-3 activation and lowered generation of caspase 3-cleaved tau fragments [132]. Recently, minocycline was shown to protect against Aβ-induced cell death and prevent fibrillization of Aβ *in vitro*[133], reduce iNOS levels [134], prevent Aβ deposition and cognitive decline in APP transgenic mice [134,135] by reducing BACE1 levels [134], inhibit neuronal death and attenuate learning and memory deficits following administration of Aβ in rats [136,137]. In addition, treatment of a tau model with minocycline resulted in reduced levels of tau phosphorylation and insoluble tau aggregates [132].

Another potential mechanism of action of minocycline has been related to the inhibition of microglial activation. Administration of minocycline in animal models of ALS attenuated the induction of the expression of M1 microglia markers during the progressive phase, whereas it did not affect the transient enhancement of expression of M2 microglia markers during the early pathogenesis phase [138]. This study suggests that minocycline may selectively inhibit the microglia polarisation to a proinflammatory state.

### Anti-TNFα

TNFα is upregulated in AD and it has been found to increase in a stage-specific manner in the APP_SWE_/PS1dE9 mouse model [139]. Interestingly, anti-TNFα treatment with the antibody against TNFα, infliximab, reduced Aβ and tau phosphorylation in transgenic mice. In addition, infliximab increased the number of CD11c-positive dendritic-like cells and the expression of CD11c, suggesting that the CD11c-positive dendritic-like cells might contribute to the infliximab-induced reduction of AD-like pathology [140].

The TNFα inhibitor thalidomide has been found to have abilities against tumour growth, angiogenesis, and inflammation. Chronic administration of thalidomide in APP23 and 3×Tg-AD mice resulted in a dramatic decrease in the activation of both astrocytes and microglia, Aβ load, plaque formation and tau phosphorylation [141,142]. Furthermore, a significant decrease in BACE1 level and activity was also found [141]. However, it is not expected that this type of treatment will be beneficial for tau pathology, according to the results published in TNFRΙ knockout mice.

## Conclusions

The advances in AD research in the last decade have brought to light that this disease is multi-faceted in nature and is linked to a variety of different functional mechanisms in the brain. That inflammatory processes play a role in AD cannot be disputed, and yet there are still many unanswered questions as to whether this is beneficial or detrimental.

The use of genetic and drug manipulation in transgenic AD mice have provided *in vivo* support to previous *in vitro* observations regarding the potential effects of inflammation on the processing of APP and the phosphorylation of tau. In this regard, enhancing inflammation has been linked with increases in Aβ generation, Aβ aggregation and tau phosphorylation. While, at first glance, data obtained in the transgenic models might suggest differential effects of immune modulation on APP and tau models, the very few studies undertaken and reported here do seem to follow a similar hypothesis that a general enhancement of immune activation in the brain increases pathology but that targeted activation of factors promoting phagocytosis and clearance of amyloid may also reduce the hyperphosphorylation of tau. On the other hand, modulation of inflammation in the 3×Tg-AD model has suggested that the alterations on tau phosphorylation can be independent of changes in Aβ levels by these inflammatory mediators.

Preclinical investigations on anti-inflammatory treatments have shown that certain drugs target these effects and potentially decrease BACE1 transcription (such as TNFα inhibitors and PPARγ activators) and increase Aβ degradation. Current research strongly suggests that targeting specific microglial phenotypes as opposed to inflammation in general will yield more promising therapeutic results. This is important in light of the different phenotypic microglial activation in different stages of the disease. Harnessing the ability of microglia to efficiently clear Aβ has significant therapeutic potential. In addition, utilising the phagocytic capabilities of infiltrating macrophages to clear Aβ, in particular targeting CCR2 in specific myeloid lineages, would be of substantial benefit. It is also worth noting that the promising effects of anti-inflammatory drugs are possibly preventive treatments and are not aimed at curing the disease.

The studies presented here also highlight the dangers of translating observations in animal studies into human studies and clinical trials. Currently available models do not accurately and fully reflect AD in humans; however, they are particularly useful at testing and predicting how certain manipulations will affect amyloid or tau deposition more specifically rather than overall disease progression. This makes it very clear that testing any potential therapies must be undertaken in a range of AD models to fully elucidate the predicted outcome in humans. Further studies assessing the potential for targeting these specific inflammatory processes, in addition to the role of astrocytes and infiltrating macrophages, are needed to elucidate more effective treatments and provide a clearer understanding of the complexities of inflammatory signalling in AD.

## Abbreviations

α1-ACT: α_1_-antichymotrypsin; Aβ: Amyloid-beta; AD: Alzheimer’s disease; APP: Amyloid precursor protein; COX: Cyclooxygenase; IFN: Interferon; IL: Interleukin; iNOS: Inducible nitric oxide synthase; LPS: Lipopolysaccharide; MyD88: Myeloid differentiation primary response protein 88; NOS: Nitric oxide synthase; NSAID: Non-steroidal anti-inflammatory drug; PPAR: Peroxisome proliferator-activated receptor; RXR: Retinoid X receptor; Scar: Scavenger receptor; Tg: Transgenic; TLR: Toll-like receptors; TNF: Tumor necrosis factor; TSPO: Translocator protein.

## Competing interests

The authors declare that they have no competing interests.

## Authors’ contributions

AMB and MS contributed equally to drafting the main text, and LK produced the tables and references. All authors read and approved the final manuscript.
